# Evaluation of the impact of genetic polymorphisms in glutathione-related genes on the association between methylmercury or n-3 polyunsaturated long chain fatty acids and risk of myocardial infarction: a case-control study

**DOI:** 10.1186/1476-069X-10-33

**Published:** 2011-04-19

**Authors:** Karin S Engström, Maria Wennberg, Ulf Strömberg, Ingvar A Bergdahl, Göran Hallmans, Jan-Håkan Jansson, Thomas Lundh, Margareta Norberg, Gerda Rentschler, Bengt Vessby, Staffan Skerfving, Karin Broberg

**Affiliations:** 1 Division of Occupational and Environmental Medicine, Lund University, Lund, Sweden; 2 Department of Medicine, Skellefteå Hospital, Skellefteå, Sweden; 3 Department of Public Health and Clinical Medicine, Umeå University, Umeå, Sweden; 4 Department of Public Health and Caring Sciences, Uppsala University, Uppsala, Sweden

**Keywords:** Methylmercury, myocardial infarction, polymorphisms, glutathione, n-3 polyunsaturated long chain fatty acids

## Abstract

**Background:**

The n-3 polyunsaturated fatty acids eicosapentaenoic acid and docosahexaenoic acid, which are present in fish, are protective against myocardial infarction. However, fish also contains methylmercury, which influences the risk of myocardial infarction, possibly by generating oxidative stress. Methylmercury is metabolized by conjugation to glutathione, which facilitates elimination. Glutathione is also an antioxidant. Individuals with certain polymorphisms in glutathione-related genes may tolerate higher exposures to methylmercury, due to faster metabolism and elimination and/or better glutathione-associated antioxidative capacity. They would thus benefit more from the protective agents in fish, such as eicosapentaenoic+docosahexaenoic acid and selenium. The objective for this study was to elucidate whether genetic polymorphisms in glutathione-related genes modify the association between eicosapentaenoic+docosahexaenoic acid or methylmercury and risk of first ever myocardial infarction.

**Methods:**

Polymorphisms in glutathione-synthesizing (glutamyl-cysteine ligase catalytic subunit, *GCLC *and glutamyl-cysteine ligase modifier subunit, *GCLM*) or glutathione-conjugating (glutathione S-transferase P, *GSTP1*) genes were genotyped in 1027 individuals from northern Sweden (458 cases of first-ever myocardial infarction and 569 matched controls). The impact of these polymorphisms on the association between erythrocyte-mercury (proxy for methylmercury) and risk of myocardial infarction, as well as between plasma eicosapentaenoic+docosahexaenoic acid and risk of myocardial infarction, was evaluated by conditional logistic regression. The effect of erythrocyte-selenium on risk of myocardial infarction was also taken into consideration.

**Results:**

There were no strong genetic modifying effects on the association between plasma eicosapentaenoic+docosahexaenoic acid or erythrocyte-mercury and risk of myocardial infarction risk. When eicosapentaenoic+docosahexaenoic acid or erythrocyte-mercury were divided into tertiles, individuals with *GCLM*-*588 TT *genotype displayed a lower risk relative to the *CC *genotype in all but one tertile; in most tertiles the odds ratio was around 0.5 for *TT*. However, there were few *TT *carriers and the results were not statistically significant. The results were similar when taking plasma eicosapentaenoic+docosahexaenoic acid, erythrocyte-selenium and erythrocyte-mercury into account simultaneously.

**Conclusions:**

No statistically significant genetic modifying effects were seen for the association between plasma eicosapentaenoic+docosahexaenoic acid or erythrocyte-mercury and risk of myocardial infarction. Still, our results indicate that the relatively rare *GCLM*-*588 TT *genotype may have an impact, but a larger study is necessary for confirmation.

## Background

The toxic compound methylmercury (MeHg) from fish may increase the risk of myocardial infarction (MI), possibly due to induction of oxidative stress, a risk factor for MI. High mercury (Hg) levels in hair (proxy for MeHg retention) [1,2] and toenails (proxy for MeHg) [3] have been associated with an increased risk of MI. However, high Hg levels in erythrocytes (Ery-Hg; proxy for MeHg) have been associated with a decreased risk of MI in some studies [4,5]. This discrepancy between studies and populations may partly be explained by different MeHg exposure levels in different studies. However, genetic differences between individuals and populations may explain some of these discrepancies; some individuals may have a genetic setup giving a more beneficial MeHg metabolism, resulting in a faster MeHg-elimination [6,7] and/or differences in capacity to detoxify reactive oxygen species induced by MeHg. Individuals with a more beneficial MeHg metabolism and/or a better capacity to detoxify reactive oxygen species may be able to tolerate higher exposures to MeHg. Subsequently, they would benefit more from protective agents in fish, the major source of MeHg exposure. Protective agents include the n-3 polyunsaturated long chain fatty acids eicosapentaenoic acid (EPA) and docosahexaenoic acid (DHA) (their sum referred to as EPA+DHA), which has antiatherogenic, antithrombotic and antiarrhythmic properties [8,9], as well as selenium (Se), which may protect against MI due to its presence in the antioxidant enzyme glutathione peroxidase.

MeHg is eliminated in the bile as a glutathione (GSH) conjugate [10,11]. The rate-limiting enzyme for GSH synthesis is glutamyl-cysteine ligase (GCL), which is composed of a catalytic subunit (GCLC [Unigene: Hs.654465] [12]) and a modifier subunit (GCLM [Unigene: Hs.315562]). The glutathione *S*-transferases (GSTs), which conjugate GSH to a wide variety of electrophilic compounds [13,14], may also affect the metabolism of MeHg. Polymorphisms with a functional impact in genes coding for GSH-synthesizing (GCLC, GCLM) or GSH-conjugating (GSTs) enzymes may thus influence the elimination capacity. Polymorphisms affecting GSH production (lower promoter activity for the variant alleles) have been found in both subunits; a *C*→*T *nucleotide substitution at position -*129 *in *GCLC *(*GCLC*-*129*) [15,16] and a *C*→*T *nucleotide substitution at position -*588 *in *GCLM *[17]. Glutathione-S-transferase pi (*GSTP1 *[Unigene [12]: Hs.523836]) exhibits a number of genetic polymorphisms, of which the Ile105Val and Ala114Val amino acid substitutions both have been associated with differences in enzyme activity and substrate preferences [18-20]. Furthermore, GSH is an antioxidative agent, and polymorphisms in *GCLC*, *GCLM *and *GSTP1 *may thus modify the MeHg-induced oxidative damage.

In our previous study [7], we evaluated the genetic impact on MeHg metabolism. This study did not include any MI cases and consisted of different study subjects than the present study. We found that carriers of the variant alleles for the *GSTP1 *polymorphisms *GSTP1*-105 or *GSTP1-114 *had lower levels of Ery-Hg at high plasma EPA+DHA, compared with individuals with no variant alleles, indicating a faster elimination of MeHg for variant-allele carriers. Also, *TT *homozygotes for the *GCLM*-588 polymorphism demonstrated higher Ery-Hg than the other *GCLM*-588 genotypes. Polymorphisms in *GCLC*, *GCLM *and/or *GSTP1 *have been associated with body burden of Hg in other studies [6,21,22].

The present study is an extension of a study by Wennberg et al [5], which evaluated the impact of P-EPA+DHA and Ery-Hg on MI risk in this study population. The study by Wennberg et al. found that Ery-Hg was statistically significantly associated with a decreased risk of MI, while EPA+DHA in plasma indicated a decreased risk of MI (not statistically significant). The aim of the present study was to elucidate whether polymorphisms in genes potentially involved in MeHg metabolism and/or antioxidant defense can modify the association between EPA+DHA or Ery-Hg and MI risk. The effect of erythrocyte-Se (Ery-Se) on MI risk was also considered.

## Methods

### Study population

The study population is part of an individually matched case-control study nested within three prospective health surveillance cohorts in Västerbotten, Northern Sweden: the Västerbotten Intervention Program (VIP), the WHO's Multinational Monitoring of Trends and Determinants in Cardiovascular Disease (MONICA) Study in northern Sweden, and the Mammography Screening Project (MSP). Both VIP and MONICA are health examination programs for CVD and diabetes. The cohorts, sampling and other variables are described more thoroughly in Wennberg et al. [5]. Medical history and lifestyle information was obtained at baseline in 1987 - 1999 (Table 1). Venous blood was sampled and erythrocytes, serum and plasma were separated and stored in a biobank at -80°C. Controls were matched to the cases for first ever MI (one control for each male case and two for each female case) for sex, age (±2 years), date of health survey (±4 months) and geographical region. Cases and controls were excluded if previous MI, stroke, or malignant disease could not be excluded according to the questionnaire or case records. Erythrocytes and DNA were available from 1027 individuals (458 cases and 569 controls); 42 cases and 56 controls were missing compared with Wennberg et al. [5], due to lack of samples for DNA extraction. Plasma was available from 916 individuals (408 cases and 508 controls). Data on smoking was missing for 39 individuals, and data on apolipoprotein B/A1 ratio (ApoB/ApoA1) was missing for 3 individuals. Smoking habits were classified into "daily smoking" or "nonsmoking" (including previous smokers and occasional smokers). The study was approved by the Regional Ethics Committee of Umeå University. Participants gave written informed consent prior to the study.

**Table 1:** Descriptive data of the study population.

	Controls				Cases			
	
	N	Mean	SD	Range	N	Mean	SD	Range
Ery-Hg (μg/l)	569	4.9	5.1	0.19-81	458	4.5	5.7	0.010-87

P-EPA+DHA (%)	508	6.2	1.6	2.8-14	408	6	1.5	3.2-15

Ery-Se (μg/l)	568	130	37	75-710	458	130	22	72-210

ApoB/apoA1	567	0.8	0.26	0.14-2.1	457	0.95	0.25	0.21-2.1

BMI (kg/m^2^)	435	26	4	18-49	378	27	4	18-57

Smokers	555	19%			433	42%		

Fish (meals/week)	427	1.3	0.79	0.0-5.5	354	1.2	0.94	0.0-8.0

-lean fish	431	0.72	0.67	0.0-6.5	365	0.68	0.58	0.0-4.0

-fat fish	433	0.57	0.47	0.0-4.0	361	0.58	0.58	0.0-4.0

*Abbreviations: *SD, standard deviation; Ery-Hg, erythrocyte total mercury concentration; Ery-Se, erythrocyte selenium concentration.

### Measurement of Ery-Hg, Ery-Se and P-EPA+DHA

The determination of Ery-Hg was made in acid-digested samples using cold vapor atomic fluorescence spectrometry [5,7,23]. Plasma fatty acids were separated by gas-liquid chromatography after separation of the lipids by thin layer chromatography and transmethylation [24]. Plasma EPA+DHA (P-EPA+DHA) was calculated as the percent in plasma phospholipids [4,25]. APOA1 and APOB were measured by immunoturbidimetry, while Ery-Se was measured by inductively coupled plasma mass spectrometry (ICP-MS; Thermo × 7; Thermo Elemental, Winsford, United Kingdom).

More detailed information, such as limit of detection, coefficients of variance, and reference material are thoroughly described in Wennberg et al. [5].

### Genotype analyses

The Taqman allelic discrimination assay (ABI 7000, Applied Biosystems, Foster City, CA, USA) was performed to analyze all four single nucleotide polymorphisms (SNPs) separately; *GCLC-129, GCLM-588, GSTP1-105 *and *GSTP1-114 *[6,7]. There was a total agreement between the original and repeat genotyping runs in all of the four SNPs analyzed. Genotype frequencies as well as the ID numbers (rs numbers [26]) for the polymorphisms are presented in Table 2.

**Table 2:** Genotype frequencies for *GCLC*-129, *GCLM*-588, *GSTP1*-105 and *GSTP1*-114.

Gene	Polymorphism	Genotype	Frequency (%)
*GCLC*	*-129C/T*	*CC*	86

*rs17883901*		*CT*	13

		*TT*	0.5

*GCLM*	*-588C/T*	*CC*	75

*rs41303970*		*CT*	22

		*TT*	2.9

*GSTP1*	*Ile105Val*	*Ile/Ile*	51

*rs1695*		*Ile/Val*	40

		*Val/Val*	8.8

*GSTP1*	*Ala114Val*	*Ala/Ala*	82

*rs1138272*		*Ala/Val*	17

		*Val/Val*	1.4

*Abbreviations: GCLC*, glutamyl-cysteine ligase catalytic subunit; *GCLM*, glutamyl-cysteine ligase modifier subunit; G*STP1*, glutathione-*S*-transferase Pi 1.

### Statistical analysis

Hardy-Weinberg equilibrium was tested for the controls using the conventional Chi-square test. Because the effects of P-EPA+DHA, Ery-Se and Ery-Hg on MI risk were in the same direction for men and women [5], both genders were combined in the multivariate analyses. Due to a lot of missing data for some of the background variables (especially for females), we only considered adjustments for age, smoking, BMI, year of sampling, and ApoB/ApoA1. In total, we employed four genetic markers (*GCLC*-129, *GCLM*-*588, GSTP1-105 *and *GSTP1*-*114*). Effects of dual-polymorphisms for the *GSTP1 *gene (*GSTP1*-*105 *and *GSTP1*-*114*) were also analyzed, where genotype was categorized as the total number of variant alleles for both polymorphisms. Individuals with two or more variant alleles were pooled into one group. The genotypes were used as categorical variables in all analyses. Since this study is an extension of the study by Wennberg et al [5], P-EPA+DHA and Ery-Hg were evaluated in a similar fashion - divided into tertiles - as in Wennberg et al [5].

**Figure 1:**
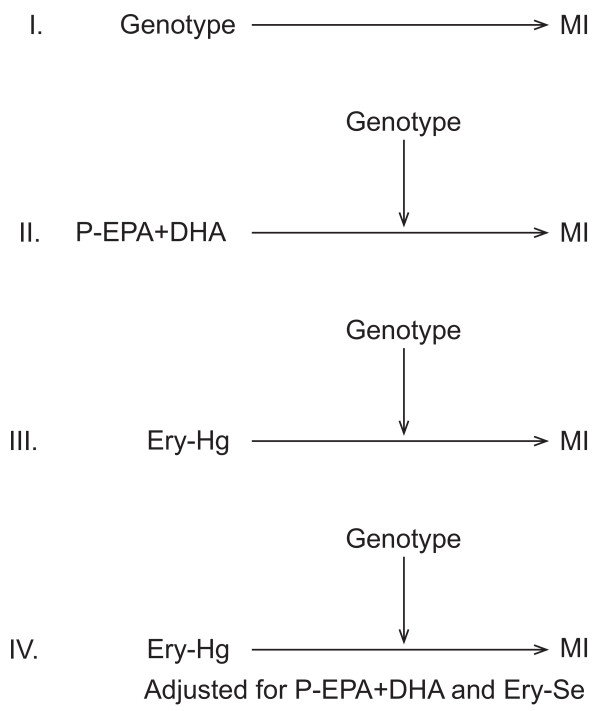
**Schematic overview of the statistical analyses performed**. *Abbreviations: *MI, myocardial infarction; Ery-Hg, erythrocyte total mercury concentration; Ery-Se, erythrocyte selenium concentration).

A schematic overview of the statistical analyses are shown in Figure 1. For all analyses, a univariate conditional logistic regression model (unadjusted model), based on the matched case-control sets was performed, as well as a multivariate logistic regression model (adjusted model), including covariates that had a p-value < 0.05.

Firstly, we evaluated whether genotype in itself had any effect on MI risk (I in Figure 1). For each polymorphism, the group with the most common homozygous genotype was used as reference. For the *GSTP1*-*105 *and *GSTP1*-*114 *combination, the group with individuals with 0 variant alleles was used as reference.

Secondly, we evaluated whether the association between P-EPA+DHA and MI risk was modified by genotype due to its potential impact on MeHg metabolism (II in Figure 1). Genetic factors may have an impact on MeHg metabolism, resulting in an individual variation regarding retention and elimination of MeHg at similar exposure levels. Differences in retention/elimination lead to differences in the amount of MeHg that has the opportunity to target vulnerable organs and cause toxic effects that may increase the risk of MI. In order to evaluate this, P-EPA+DHA can be used as a proxy for intake of MeHg, since P-EPA+DHA correlates with the body burden of MeHg. P-EPA+DHA was stratified into three groups, according to tertiles in the controls (0-5.39, 5.40-6.68, and 6.69-15%, respectively). A univariate and a multivariate conditional logistic regression model were run for each stratum. The group consisting of the most common homozygotes at the lowest P-EPA+DHA tertile was used as reference.

Thirdly, we evaluated whether the association between Ery-Hg and MI risk was modified by genotype, due to its potential role in protection against oxidative stress (III in Figure 1). Ery-Hg was stratified according to tertiles in the controls (0-2.81, 2.82-4.79, and 4.83-87 μg/l, respectively). The tertile boundaries differed somewhat from Wennberg et al. [5], due to a different number of individuals. A univariate and multivariate conditional logistic regression model were run for each stratum. The group consisting of the most common homozygotes at the lowest Ery-Hg tertile was used as reference.

Fourthly, we evaluated whether the effect of Ery-Hg on MI risk was modified by genotype (IV in Figure 1), when adjustments were done for P-EPA+DHA and Ery-Se.

Because of the low allelic frequencies and similar effects of some genotypes, the heterozygotes and homozygous variant genotypes for the same gene were pooled in all analyses for *GCLC*-*129 *and *GSTP1*-*114*.

All statistical analyses were performed using the software SPSS (version 15; SPSS, Chicago, IL, USA). All tests were two-sided and p ≤ 0.05 was considered statistically significant.

## Results

When genotype was not taken into account, P-EPA+DHA indicated a decreasing MI risk (p = 0.15), while Ery-Hg was statistically significantly associated with decreasing MI risk (p = 0.014) (Wald test for univariate analyses for the trichotomized biomarkers).

There were significant effects on MI risk by smoking (OR 3.2, 95% CI 2.4, 4.5) and ApoB/ApoA1 (OR 12.7, 95% CI 6.7, 24) and these two variables were thus included in the multivariate analyses.

All polymorphisms were in Hardy-Weinberg equilibrium (analyzed for controls only) except for *GCLM*-*588 *(p = 0.030).

When evaluating the effect of genotype alone on MI risk (I in Figure 1), no significant results were seen. For example, the adjusted odds ratio (OR) for the *GCLM-588 TT *genotype (*GCLM-588 CC *was used as reference) was 0.60 (95% CI 0.24, 1.5).

**Figure 2:**
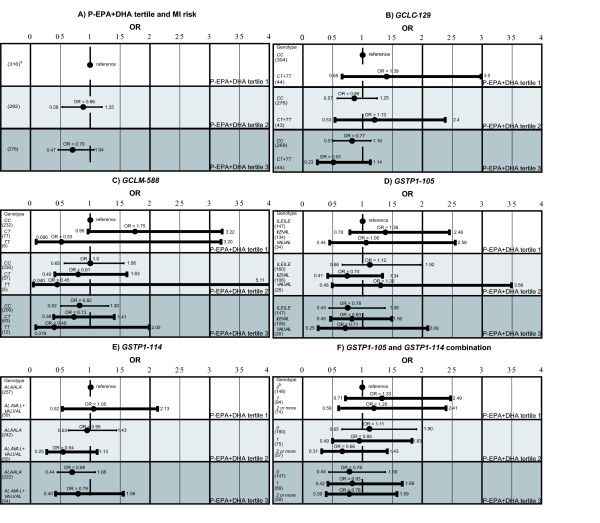
**Genetic impact on the association between P-EPA+DHA and MI risk**. Risk of MI (Odds Ratio, OR) with 95% confidence intervals (CI) for different genotypes in the P-EPA+DHA tertiles. The reference group is the most common homozygote in the lowest P-EPA+DHA tertile. ORs are adjusted for smoking and ApoB/ApoA1 ratio. The tertile boundaries are 0-5.39, 5.40-6.68, and 6.69-15%. ^a ^Number of individuals in each group.^b ^Number of variant alleles.

**Figure 3:**
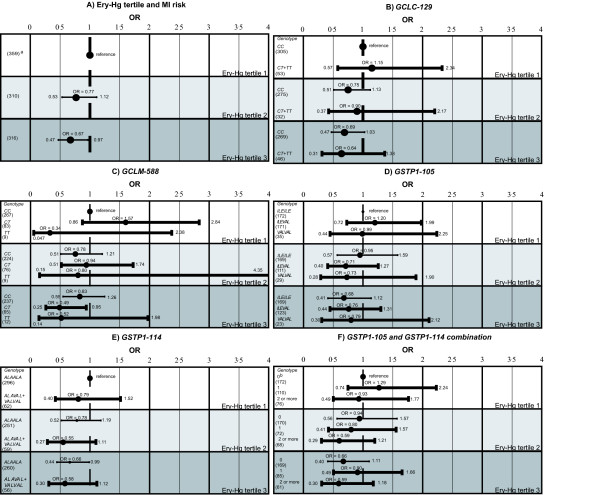
**Genetic impact on the association between Ery-Hg and MI risk**. Risk of MI (OR) with 95% CI for different genotypes in the Ery-Hg tertiles. The reference group is the most common homozygote in the lowest Ery-Hg tertile. ORs are adjusted for smoking and ApoB/ApoA1 ratio. The tertile boundaries are 0-2.81, 2.82-4.79, and 4.83-87 μg/l. ^a ^Number of individuals in each group. ^b^Number of variant alleles.

There were no statistically significant genetic modifying effect of the associations between P-EPA+DHA or Ery-Hg and MI risk (Figures 2 and 3). The variant homozygotes (*TT*) for the *GCLM-588 *polymorphism had a lower MI risk relative to wildtype homozygotes (*CC*) in all P-EPA+DHA and Ery-Hg tertiles (Figures 2, graph C and 3, graph C). The ORs for *TT *were around 0.5 in most tertiles. However, this was not statistically significant and it was based on few individuals with the *TT *genotype. Among the controls, the *GCLM TT *carriers demonstrated a mean Ery-Hg level of 5.4 μg/l among the controls compared to 4.9 μg/l among the *GCLM CC+CT *carriers (p-value = 0.65, ANOVA).

For *GSTP1 *(combination of *GSTP1-105 *and *GSTP1-114*), carriers of two or more variant alleles showed the lowest risk in all Ery-Hg tertiles (Figure 3, graph F). However, the differences were not statistically significant.

All analyses gave similar genetic modifying effects on the association between Ery-Hg and MI risk when adjustments for P-EPA+DHA and Ery-Se were done compared with when no adjustments for P-EPA+DHA and Ery-Se were done (data not given here).

## Discussion

Similar patterns as in Wennberg et al. [5] were seen regarding the associations between Ery-Hg and P-EPA+DHA, respectively, and MI risk. This is not surprising, since the study populations are, largely, the same in both studies. It is also in accordance with a previous, smaller study in the same region [4]. The protective effect of P-EPA+DHA is in concordance with numerous other studies. The association between high Ery-Hg and low MI risk is most likely because Ery-Hg is a marker of fish intake.

We found that the polymorphisms studied had no effect on MI risk by themselves. When P-EPA+DHA or/and Ery-Hg was taken into account, the *GCLM-588 TT *genotype had a lower risk relative to the *CC *genotype in all P-EPA+DHA or Ery-Hg tertiles except for one (intermediate tertile for Ery-Hg) (ORs generally around 0.5), but these findings were not statistically significant. The lack of statistically significant results in this study may be due to a small number of individuals for some genotypes, in particular *GCLM-588 TT*, yielding a too low power to detect statistically significant effects. The effect estimates (ORs) for *GCLM*-*588 TT *were strongly deviant from the *CT *genotype, which prevented us from pooling these two genotypes. In our previous study, the *GCLM-588 TT *genotype demonstrated the highest levels of Ery-Hg [7]. The *GCLM-588 TT *genotype had the highest levels of Ery-Hg also among the controls in this study, although this was not significant. *GCLM-588 TT *carriers displayed the lowest risk of MI in each P-EPA+DHA tertile in this study. However, it is important to note that this interpretation is based on few individuals and needs to be confirmed.

The *GCLM-588 *was in Hardy-Weinberg disequilibrium. The allele frequencies reported here were similar to another study, analyzing Danish and Swiss populations, where Hardy-Weinberg equilibrium was reported [27]. The preceding gene-environment studies for MeHg with individuals from the same geographical area [6,7] also showed linkage disequilibrium, while we observed equilibrium in other studies by our group, employing the same methods, but in other populations [21,28]. Therefore, the linkage disequilibrium may be population-specific.

To our knowledge, no other studies have assessed gene-environment interactions (for any gene) on the association between MeHg or n-3 polyunsaturated fatty acids and MI risk. There may, however, also be other genetic traits that can influence these associations, e.g. polymorphisms in other GSH-related genes, such as glutathione peroxidase *(GPX1)*, glutaredoxin *(GLRX)*, gamma-glutamyl transferase 1 *(GGT1)*, or in any of the other *GSTs*. It is also possible that thioredoxin *(TXN) *and *TXN*-related genes affect the metabolism of MeHg [29]. In addition to the genes we studied, there may be an influence of other genes involved in oxidative stress, such as catalase *(CAT)*, paroxonases *(PONs) *and superoxide dismutase *(SOD)*.

A number of studies have investigated the genetic impact on MI risk. Some included genes potentially involved in protection against oxidative stress: the *GCLM-588 T*-allele has been associated with a higher risk of MI (non-lethal cases [17]). The T-allele showed a lower promoter activity in response to oxidants, and the increase of mRNA expression in response to oxidants was significantly lower among *CT *subjects, compared to *CC *subjects. The variant allele of *GCLC-129 *has also been associated with MI [16]. However, none of these findings was confirmed in our study.

## Conclusions

There was no statistically significant genetic modifying impact on the association between P-EPA+DHA or Ery-Hg and MI risk. Still, our results indicate that the relatively rare *GCLM*-588 TT genotype may have an impact, but a larger study is necessary for testing this hypothesis.

## List of abbreviations

ApoB/A1: Apolipoprotein B/A1 ratio; BMI: Body mass index; CI: Confidence interval; DHA: Docosahexaenoic acid; EPA: Eicosapentaenoic acid; Ery-Hg: Erythrocyte total mercury concentration; Ery-Se: Erythrocyte selenium concentration; GCL: Glutamyl-cysteine ligase; *GCLC*: Glutamyl-cysteine ligase catalytic subunit; *GCLM*: Glutamyl-cysteine ligase modifier subunit; GSH: Glutathione; GST: Glutathione-*S*-transferase; *GSTP1*: Glutathione-*S*-transferase Pi 1; Hg: Mercury; MeHg: Methylmercury; MI: Myocardial infarction; OR: Odds ratio; SD: Standard deviation; Se: Selenium; SNP: Single nucleotide polymorphism.

## Competing interests

The authors declare that they have no competing interests.

## Authors' contributions

KSE. contributed to data preparation, designed and carried out the data analyses, participated in interpretation of data, drafting and finalizing of the manuscript. MW contributed to data preparation, participated in interpretation of data, drafting and finalizing of the manuscript. US was advisor for the statistical analyses, contributed to interpretation of data, drafting and finalizing of the manuscript. IAB participated with input in the manuscript writing and analysis of the data. GH participated in the discussion initiating the study, collection and administration of data. TL carried out chemical analysis of Ery-Hg and Ery-Se. MN participated in the discussion initiating the study, collection and administration of data. GR performed genotyping analyses. BV carried out chemical analysis of fatty acids. SS participated in the discussion initiating the study, participated in data analyses, interpretation of data and drafting and finalizing of the manuscript. JHJ established the case-control study population. KB participated in the discussion initiating the study, participated in data analyses, interpretation of data and drafting and finalizing of the manuscript. All authors have read and approved the final manuscript.
